# Prediction of conversion to dementia disorders based on timed up and go dual-task test verbal and motor outcomes: a five-year prospective memory-clinic-based study

**DOI:** 10.1186/s12877-023-04262-w

**Published:** 2023-09-02

**Authors:** Anna Cristina Åberg, Johanna R. Petersson, Vilmantas Giedraitis, Kevin J. McKee, Erik Rosendahl, Kjartan Halvorsen, Lars Berglund

**Affiliations:** 1 School of Health and Welfare, Dalarna University, 791 88 Falun, Sweden; 2 Department of Public Health and Caring Sciences, Geriatrics, Uppsala Universit, y, Box 564, 52 37 UPPSALA, Sweden; 3 Department of Community Medicine and Rehabilitation, Physiotherapy, Umeå University, 90187 Umeå, Sweden; 4 Department of Mechatronics, School of Engineering and Sciences, Campus Estado de Mexico, Tecnologico de Monterrey, Atizapan, Mexico, Carretera Lago de Guadalupe Km 3.5, 52926 Atizapan, Estado de Mexico, Mexico

**Keywords:** Mild cognitive impairment, Subjective cognitive impairment, Demenetia disorder, Dual-task, Gait

## Abstract

**Background:**

While assessment tools can increase the detection of cognitive impairment, there is currently insufficient evidence regarding clinical outcomes based on screening for cognitive impairment in older adults.

**Methods:**

The study purpose was to investigate whether Timed Up and Go dual-task test (TUGdt) results, based on TUG combined with two different verbal tasks (name different animals, TUGdt-NA, and recite months in reverse order, TUGdt-MB), predicted dementia incidence over a period of five years among patients (*N* = 186, mean = 70.7 years; 45.7% female) diagnosed with Subjective Cognitive Impairment (SCI) and Mild Cognitive Impairment (MCI) following assessment at two memory clinics. Associations between TUG parameters and dementia incidence were examined in Cox regression models.

**Results:**

During follow-up time (median (range) 3.7 (0.1–6.1) years) 98 participants converted to dementia. Novel findings indicated that the TUGdt parameter words/time, after adjustment for age, gender, and education, can be used for the prediction of conversion to dementia in participants with SCI or MCI over a period of five years. Among the TUG-related parameters investigated, words/time showed the best predictive capacity, while time scores of TUG and TUGdt as well as TUGdt cost did not produce significant predictive results. Results further showed that the step parameter step length during TUGdt predicts conversion to dementia before adjustment for age, gender, and education. Optimal TUGdt cutoffs for predicting dementia at 2- and 4-year follow-up based on words/time were calculated. The sensitivity of the TUGdt cutoffs was high at 2-year follow-up: TUGdt-NA words/time, 0.79; TUGdt-MB words/time, 0.71; reducing respectively to 0.64 and 0.65 at 4-year follow-up.

**Conclusions:**

TUGdt words/time parameters have potential as cost-efficient tools for conversion-to-dementia risk assessment, useful for research and clinical purposes. These parameters may be able to bridge the gap of insufficient evidence for such clinical outcomes.

**Trial registration:**

ClinicalTrials.gov Identifier: NCT05893524: https://www.clinicaltrials.gov/study/NCT05893524?id=NCT05893524&rank=1.

**Supplementary Information:**

The online version contains supplementary material available at 10.1186/s12877-023-04262-w.

## Introduction

The number of people with dementia is estimated to increase from 57.4 million globally in 2019 to 152.8 million in 2050 [1]. Cognitive impairments that may be forerunners of dementia are Subjective Cognitive Impairment (SCI) and Mild Cognitive Impairment (MCI), which can remain stable for decades, disappear, or entail further cognitive decline [2]. Although dementia disorders and cognitive impairment are growing threats to public health, [3] causing high rates of disability and social costs, they are frequently underdiagnosed for several reasons [4, 5]. This is problematic since detection is a prerequisite for health promotion intervention and planning for future care and treatment. However, while assessment tools can increase the detection of cognitive impairment there is currently insufficient evidence regarding clinical outcomes based on screening for cognitive impairment in older adults [6].

Dementia is characterized by cognitive and motor skills impairment with a gradual deterioration linked to cognition, mobility and gait, which can be explained by the shared neuroanatomical structures and processes relating to such functioning [7, 8]. Consequently, dual cognitive and mobility impairment are associated with an increased risk of developing dementia disorders [9]. Dual-task (dt) testing that challenges attentional capacities by the simultaneous performance of two tasks, commonly including straight line gait and a verbal task, may produce outcomes indicative of cognitive capacity. Such testing has been suggested as a method for identifying and predicting dementia disorders [10, 11]. Recent research examining mobility and cognitive function has shown that dual decline is associated with a higher dementia risk than memory or gait decline alone [7, 12, 13]. A relatively complex mobility task such as the Timed Up and Go test (TUG) [14], which includes both walking and transitions and places demands on executive functions [15], may in combination with a verbal task be assumed to create greater interference than straight-line walking combined with a similar task. In previous dt studies involving TUG, time scores, qualitative evaluation and quantification of correct/incorrect performance have been investigated, focusing on cognitive status and fall history [16–19]. To enable detailed assessments of simultaneous performance of both the involved tasks, video recording can be a valuable tool [19].

In the longitudinal Uppsala-Dalarna Dementia and Gait project (UDDGait™) we explore Timed Up and Go dual-task (TUGdt) testing through systematic analyses of video-recorded tests in a clinical setting [20]. TUGdt comprises TUG combined with two different verbal tasks (name different animals, TUGdt-NA, and recite months in reverse order, TUGdt-MB), targeting semantic memory and executive function, [21, 22] to provide an assessment method for dual cognitive and mobility impairment and risk of dual decline. To capture both motor and verbal performance of the TUGdt tests, results presented so far have been based on parameters derived from the number of correct words per 10 s of the TUGdt performance (TUGdt words/time). The results indicated an association between TUGdt words/time performance and neurodegeneration [23]. Additionally, they showed that TUGdt words/time demonstrates high levels of discrimination between healthy controls, SCI, MCI and dementia, and that TUGdt predicts conversion to dementia from SCI and MCI over a period of 2.5 years, most accurately among young-old adults (< 72 years) [24].

Based on UDDGait™ data, the primary purpose of this study is to investigate whether TUGdt test results predict dementia incidence among patients with SCI or MCI over a period of five years. Secondary objectives are first, to calculate the optimal prognostic cutoff values for TUGdt words/time results, and second, to determine whether TUGdt step parameters extracted from video recordings give incremental prediction above TUGdt words/time results.

## Methods

### Design, setting and participants

**Fig. 1:**
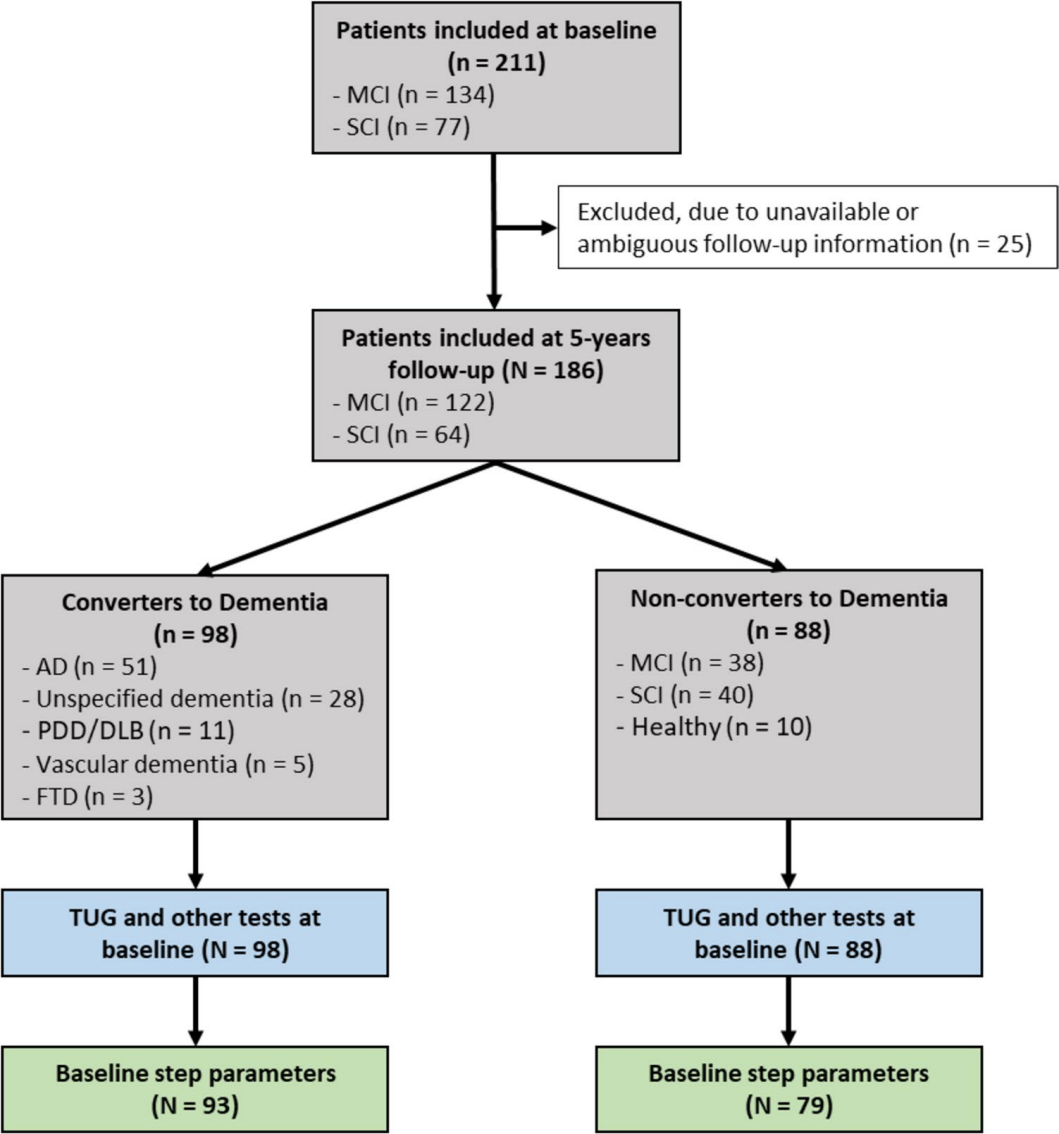
Flowchart for the inclusion and procedures for testing and data processing. Grey squares shows clinical data, blue and green—data collection and preparation, respectively. MCI – Mild Cognitive Impairment; SCI – Subjective Cognitive Impairment; AD – Alzheimer’s disease; PDD—Parkinson's disease dementia; DLB—dementia with Lewy bodies; FTD—frontotemporal dementia; TUG—Timed Up and Go

The current prospective memory-clinic-based study forms part of the UDDGait [20], an ongoing, longitudinal study, in which patients have been consecutively included when undergoing memory assessment at two memory-clinics in Sweden during the period 2015–2017 (Fig. 1). Patients were referred to those clinics by a family physician or booked an appointment themselves.

Participants who were diagnosed with SCI or MCI at baseline were included in the current study (Table 1)). The MCI group consisted mainly of amnestic MCI patients. The baseline exclusion criteria were: inability to walk three meters back and forth or to rise from a sitting position, indoor use of a walking aid, current or recent (within the previous two weeks) hospitalization, or need of an interpreter to communicate in Swedish. Due to the insufficient follow-up information about cognitive health 25 individuals were excluded from the analysis, four of whom had died before 2-year follow-up. Ethical approval was granted from the Regional Ethical Review Board in Uppsala. Informed consent was attained from all participants.

**Table 1:** Participant characteristics and test results at baseline in total sample and stratified by conversion to dementia

**Characteristic**	**Total analyzed sample** **(*n* = 186)**	**Converters to dementia** **(*n* = 98)**	**Non-converters** **to dementia** **(*n* = 88)**	
Age, years, mean ± SD (min–max)	70.7 ± 9.1 (39–91)	74.2 ± 7.4 (56–91)	66.9 ± 9.2 (39–91)	
Female, n (%)	85 (45.7)	51 (52.0)	34 (38.6)	
University education, n (%)	81 (43.5)	39 (39.8)	42 (47.7)	
Married or cohabiting, n (%)	122 (65.6)	60 (61.2)	62 (70.1)	
Follow-up time, years, median (min–max)	3.7 (0.1–6.1)	2.3 (0.1–6.1)	4.0 (0.1–5.5)	
**Test Result**				Standardized difference
MMSE score	27.5 (25–29)	26 (24–28)	28 (27–29)	0.80
Clock drawing score	7.0 (6.0–7.0)	6.0 (5.0–7.0)	7.0 (7.0–7.0)	0.39
Verbal fluency score	17.0 (13.0–23.0)	15.5 (12.0–19.0)	20.0 (15.0–25.0)	0.59
Depressive symptoms, n (%)^a^	45 (24.3)	21 (21.4)	24 (27.6)	-
TUG, s	12.0 (10.3–14.2)	12.8 (11.2–15.0)	11.3 (9.7–12.8)	0.37
TUGdt-NA, s	13.3 (11.4–15.9)	14.1 (12.2–17.5)	12.0 (10.8–14.4)	0.49
TUGdt-NA cost, %	11.6 (3.6–18.3)	12.2 (3.9–21.3)	11.1 (3.5–14.9)	0.10
TUGdt-NA, number of animals	6.0 (5.0–8.0)	6.0 (5.0–7.8)	7.0 (6.0–8.0)	0.46
TUGdt-NA, animals/10 s	4.6 (3.3–6.1)	3.8 (2.8–5.1)	5.4 (4.2–6.4)	0.83
TUGdt-MB, s	13.5 (11.8–16.5)	14.8 (12.6–18.1)**	12.6 (10.9–15.2)	0.46
TUGdt-MB cost, %	14.2 (3.4–27.1)	18.7 (4.3–28.5)**	11.3 (3.1–25.2)	0.55
TUGdt-MB, number of months	7.0 (4.0–9.0)	6.0 (3.8–8.3)**	7.5 (6.0–9.0)	0.30
TUGdt-MB, months/10 s	4.8 (2.9–6.5)	4.1 (2.2–5.7)**	5.5 (3.9–7.7)	0.54

Baseline characteristics and test results are presented as medians and interquartile range if not stated otherwise

*SD* Standard deviation, *MMSE* Mini Mental State Examination, *TUG* Timed Up and Go, *TUGdt* Timed Up and Go dual-task, *NA* Naming animals, *MB* Months backwards Standardized differences between converters and non-converters to dementia were estimated with a non-parametric method [41]

^a^Depressive symptoms defined as two points or more on the 4-item Geriatric Depression Scale (data were not available for one participant)

^**^Only 96 from 98 individuals in this group completed TUGdt-MB test

## Data collection

### Data collection at baseline

The data collection procedures used have been described in detail previously [24, 25]. Reported demographic characteristics included educational level (university education or not) and civil status. The TUG tests were performed according to a standardized procedure and documented by video recordings of movement and speech. For descriptive purposes, participants went through a screening test using the 4-item Geriatric Depression Scale, [26] motor function assessment by a short version of the original General Motor Function Assessment Scale, [27] a balance test according to Bohannon, [28] and assessment of handgrip strength using a dynamometer [29]. The baseline data collection was carried out in connection with the initial memory assessment, which included the following routinely used cognitive tests: Mini Mental State Examination (MMSE), [30] Clock Drawing Test, [31] Verbal Fluency Test (animals per minute) [32], and Trail Making Test A and B [33]. The memory assessment resulted in a diagnosis made by a geriatrician based on established criteria.

### Timed Up and Go single- and dual-task tests at baseline

TUG involves the participant rising from an armchair, walking three meters at a comfortable pace, turning around at a mark on the floor, walking back, and sitting down again [14]. The test was timed from the participant’s back leaving the backrest to the posterior touching the seat of the chair again, and time scores for the total performance of each TUG and TUGdt test was noted. TUG is well established for the assessment of gait and mobility, does not suffer from any ceiling or floor effects in healthy older adults, [15] and has been shown to possess good reliability for people with and without disabilities across a lifespan [34].

The TUG testing for the current study was performed in the following order: TUG, TUGdt-NA, and TUGdt-MB. Participants received standardized instructions before each test from the physical therapist who led the testing procedures. For TUGdt-NA, the participant was asked to name different animals while completing the movement sequence. For TUGdt-MB, the participant was asked to recite months in reverse order, starting with December. The participants were instructed to complete all TUG tests at their own speed, concerning both mobility and verbal performance, and to complete the mobility sequence even if they did not know what to say.

### Medical records review for follow-ups

To obtain diagnostic information for follow-ups and eventual dates of death, all participants’ medical records were reviewed up to 6.1 years after baseline by an experienced geriatrician. Participants were classified as having “converted” (*n* = 98) after receiving dementia diagnosis, and as “not converted” when a diagnosis of SCI or MCI had been confirmed after baseline, or when a reversion to normal cognition had been stated, based on established criteria [35–37]. Among participants whose medical records did not provide sufficient information, MMSE score from the latest follow-up visit (within 6.1 years from base line) was used to rule out conversion to dementia, but not for establishing a dementia diagnosis. A score that was higher, unchanged, or a maximum of one point less compared with baseline, was considered to signify non-conversion. In total 88 participants were identified as non-converters, of whom 36 were identified by MMSE data. For participants with dementia, diagnosis date was noted. For one participant diagnosis date was not available, therefore the date of the last journal review was used instead. For non-converters the date of the last hospital visit or date of the last investigation was used.

### Data preparation

For TUGdt-NA and TUGdt-MB the number of correct words mentioned per 10 s during the test performance was calculated and documented as TUGdt-NA or TUGdt-MB words/time, with the time limit of a finished TUG mobility sequence, i.e. 10*(TUGdt number of correct words/TUGdt time score). Quantification of correct words recited during TUGdt-NA and TUGdt-MB was performed by reviewing the video recordings and followed the procedures used in establishing norms for such tests. For TUGdt-NA, both naming an animal group (e.g. fish) and a specific animal (e.g. salmon) were accepted [32]. For TUGdt-MB, the number of correct months in correct order was counted. A month was classified as correct when the participant started with December and then recited months in the correct order relative to the month said previously, with permission to repeat, but not to omit or transpose the months [38]. Dual-task cost was calculated as 100*(TUGdt time score –TUG time score)/TUG time score [39].

Data processing for the gait parameters was based on the documentation from two synchronized high-definition video cameras using a semi-automatic method aided by a technique for human two-dimensional pose estimation based on a deep learning procedure, described in more detail elsewhere [40] (see Supplementary material).

### Data analyses

Continuous variables were described by means and standard deviations or medians with interquartile ranges and categorical variables were described by numbers and percentages. For each participant and TUG condition, the mean value of all recorded steps was calculated for each step parameter, i.e. step length, step width, step duration and durations of single and double stance. These mean values were used in the statistical description and analysis. Standardized differences between converters and non-converters to dementia were estimated using a method for non-parametric data [41] analogous to the parametric method Cohen’s d for parametric data.

In Cox proportional hazards regression models we examined if TUG test outcomes measured at baseline examination were risk factors for incident dementia diagnoses. Univariate models were used to calculate hazard ratio (HR) for the separate TUG test outcomes and dementia incidence at follow-up (Model 1). The covariates age (continuous variable), gender, and educational level were added (Model 2). Further, to examine if step parameters give incremental prediction above TUGdt words/time parameters we estimated models where step parameters were added to TUGdt-NA or TUGdt-MB words/time and age, gender and education (Model 3). Estimated models were presented with standardized Hazard Ratio (sHR) with 95% confidence intervals (CI) and *p* values. The sHRs express the increase of hazard per one standard deviation increase of the variable, except for number of animals and months, and “animals/10 s” and “months/10 s” where the sHRs express the decrease of the variable.

Interactions between TUG test outcomes and age and sex were tested in Cox proportional hazards regression models including the above-mentioned covariates. We confirmed the proportional-hazards assumptions of the Cox models with plots of log(-log(survival)) versus log of survival time.

To explore the putative effects of reverse causation, we conducted sensitivity analyses for TUGdt words/time parameters where patients with a shorter follow-up time than one year were excluded.

In Kaplan–Meier curves, disease-free survival was displayed for participants above and below median value of number of months/10 s and number of animals/10 s.

For follow-up times of up to 2 years and up to 4 years we determined optimal cutoff values with associated prognostic diagnostic accuracy measures specificity, sensitivity and Youden index (= specificity + sensitivity – 1). Youden index is used to summarize the performance of diagnostic tests. These measures were presented with 95% CI calculated as Bootstrap percentile intervals. The 2-year time limit represents short term follow-up whereas the 4-year limit was chosen as a long term follow-up which maintained a sufficient number of participants at risk. To compare words/time with standard cognitive tests we made additional analyses where we calculated prognostic diagnostic accuracy measures for MMSE and verbal fluency tests.

All statistical tests and confidence intervals were two-sided.

Multiple test correction methods are debatable due to problems with increased Type II errors and therefore we have avoided Bonferroni correction which assumes independent tests and thus is overly conservative. We used the method of number of effective tests, which is calculated in a principal components analysis [42]. This method does take the correlation between variables into account but does not correct for estimates of both unadjusted and adjusted effects. We used 24 predictor variables and we calculated that there were 8 independent dimensions among these variables. Thus, results with *p* values < 0.05 and *p* values < 0.05/8 = 0.0062 were considered statistically significant without and with correction for multiplicity, respectively.

The statistical analyses were performed with the statistical program package SAS version 9.4 (SAS Institute Inc., Cary, NC, USA).

## Results

### Participant characteristics

The 186 participants were aged between 39 and 91 years at baseline (mean 70.7 years; 45.7% female). Of these, 64 participants were diagnosed with SCI and 122 with MCI (Fig. 1). Baseline characteristics and cognitive test results in the total sample, as well as stratified according to conversion to dementia, are summarized in Table 1. All participants completed TUGdt-NA, whereas two participants discontinued TUGdt-MB. Data for step parameters (see Fig. 1) were available for 172 participants.

### Prediction of dementia incidence

At follow-up, a total of 98 participants (53%) had converted to dementia, of which 88 had MCI and 10 had SCI at baseline. The median (range) follow-up time was 3.7 (0.1–6.1) years.

During this period five non-converter participants died. Table 2 shows estimated associations of TUG parameters with dementia incidence in Cox regression unadjusted models (Model 1) and models adjusted for age, gender, and educational level (Model 2) analyses. In Model 2 TUGdt-NA number of animals (sHR 1.24, 95% CI 1.01–1.51, *p* = 0.040), TUGdt-NA words/time (sHR 1.36, 95% CI 1.07–1.74, *p* = 0.012), TUGdt-MB number of months (sHR 1.31, 95% CI 1.06–1.61, *p* = 0.012) and TUGdt-MB words/time (sHR 1.39, 95% CI 1.10–1.75, *p* = 0.006) were significant.

**Table 2:** Hazard ratios for conversion to dementia up to 6.1 years after baseline

**Parameter**	**Model 1**		**Model 2**	
	**sHR (95% CI)**	***p*-value**	**sHR (95% CI)**	***p*-value**
TUG, s	**1.46 (1.22–1.75)**	** < 0.001**^**§**^	1.13 (0.91–1.42)	0.271
TUGdt-NA, s	**1.50 (1.25–1.80)**	** < 0.001**^**§**^	1.17 (0.92–1.47)	0.196
TUGdt-NA cost, %	1.12 (0.85–1.48)	0.419	0.99 (0.78–1.26)	0.956
TUGdt-NA, number of animals^b^	**1.24 (1.02–1.52)**	**0.035**	**1.24 (1.01–1.51)**	**0.040**
TUGdt-NA, animals/10 s^b^	**1.61 (1.30–2.00)**	** < 0.001**^**§**^	**1.36 (1.07–1.74)**	**0.012**
TUGdt-MB, s	**1.42 (1.18–1.69)**	** < 0.001**^**§**^	1.10 (0.88–1.37)	0.391
TUGdt-MB cost, %	1.12 (0.88–1.42)	0.363	0.95 (0.76–1.19)	0.662
TUGdt-MB, number of months^b^	**1.26 (1.02–1.55)**	**0.031**	**1.31 (1.06–1.61)**	**0.012**
TUGdt-MB, months/10 s^b^	**1.55 (1.24–1.93)**	** < 0.001**^**§**^	**1.39 (1.10–1.75)**	**0.006**^**§**^
TUG double stance duration, s	**1.32 (1.07–1.62)**	**0.009**	1.10 (0.88–1.37)	0.394
TUG single stance duration, s	0.98 (0.79–1.21)	0.836	1.14 (0.90–1.43)	0.282
TUG step duration, s	1.19 (0.95–1.48)	0.130	1.17 (0.93–1.48)	0.179
TUG step length, m^b^	**1.50 (1.24–1.81)**	** < 0.001**^**§**^	1.17 (0.92–1.49)	0.209
TUG step width, m	1.02 (0.82–1.26)	0.856	1.07 (0.85–1.33)	0.573
TUGdt-NA double stance duration, s	**1.28 (1.06–1.56)**	**0.012**	1.06 (0.85–1.31)	0.606
TUGdt-NA single stance duration, s	1.03 (0.83–1.28)	0.805	1.06 (0.87–1.29)	0.554
TUGdt-NA step duration, s	1.18 (0.97–1.45)	0.100	1.06 (0.87–1.29)	0.576
TUGdt-NA step length, m^b^	**1.53 (1.25–1.86)**	** < 0.001**^**§**^	1.17 (0.90–1.53)	0.238
TUGdt-NA step width, m	1.01 (0.81–1.25)	0.931	0.99 (0.78–1.27)	0.964
TUGdt-MB double stance duration, s	**1.27 (1.05–1.55)**	**0.016**	1.08 (0.87–1.36)	0.479
TUGdt-MB single stance duration, s	1.04 (0.86–1.27)	0.685	1.13 (0.93–1.38)	0.219
TUGdt-MB step duration, s	1.16 (0.97–1.39)	0.099	1.13 (0.92–1.37)	0.241
TUGdt-MB step length, m^b^	**1.50 (1.26–1.79)**	** < 0.001**^**§**^	1.19 (0.94–1.51)	0.149
TUGdt-MB step width, m	0.96 (0.76–1.20)	0.697	0.94 (0.74–1.20)	0.644

*TUG* Timed Up and Go, *TUGdt* Timed Up and Go dual-task, *NA* Naming animals, *MB* Months backwards, *sHR* Standardized Hazard Ratio, *CI* Confidence interval

sHR measure risk increase per one standard deviation *increase* of the predictor

^b^sHR measure risk increase per one standard deviation *decrease* of the predictor

Model 1: unadjusted. Model 2: adjusted for age, gender, and educational level. Statistically significant if *p* < 0.05, in bold. § Statistically significant after multiple test correction, *p* < 0.0062

In sensitivity analyses where patients with a shorter follow-up time than one year were excluded, results from adjusted models (Model 2) were TUGdt-NA words/time (sHR 1.32, 95% CI 1.01–1.72, *p* = 0.045), and TUGdt-MB words/time (sHR 1.37, 95% CI 1.06–1.75, *p* = 0.015).

In Model1 the step parameters double stance duration single task (sHR 1.32 95% CI 1.07–1.62, *p* = 0.009), step length single task (sHR 1.50, 95% CI 1.24–1.81, *p* < 0.001), double stance duration animals (sHR 1.28 95% CI 1.06–1.56, *p* = 0.012, step length animals (sHR 1.53 95% CI 1.25–1.86, *p* < 001) and step length months (sHR 1.50 95% CI 1.26–1.79, *p* < 001) were associated with dementia incidence but these associations were not significant in adjusted models (Model 2).

After multiple test correction, results for time TUG single task, time TUGdt-NA, TUGdt-NA words/time, time TUGdt-MB, TUGdt-MB words/time, step length single task, step length animals and step length months were statistically significant in unadjusted models, while TUGdt-MB words/time remained statistically significant in the adjusted model.

When step parameters were added to TUGdt-NA words/time or TUGdt-MB words/time and age, gender and education there were no significant effects of the step parameters (Model 3, data not shown). No significant interactions between TUG test outcomes and age and sex were observed. However, for TUGdt-NA word/time the p-value for interaction with age was 0.06.

**Fig. 2:**
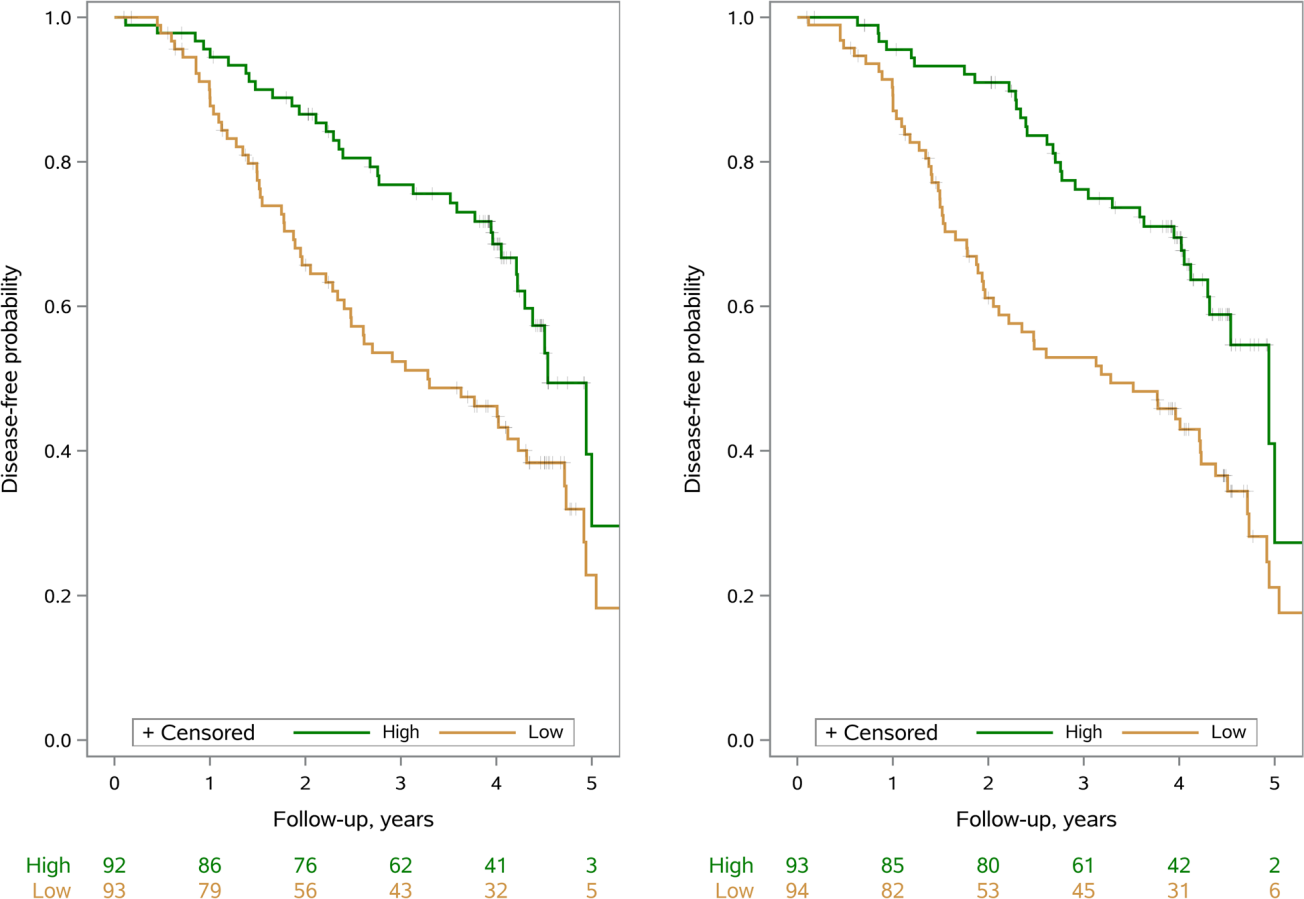
Kaplan–Meier disease-free survival curves and number at risk table: patients above (High) and below (Low), words/time median value of TUGdt-MB (left) and TUGdt-NA (right)

In Kaplan–Meier curves (Fig. 2), disease-free survival is presented for participants above (High) and below (Low) median value of TUGdt-MB word/time (left) and TUGdt-NA word/time (right). In both figures, a relatively large separation between high and low groups was observed until approximately 3 years after which they converge.

### Prognostic diagnostic accuracy

**Fig. 3:**
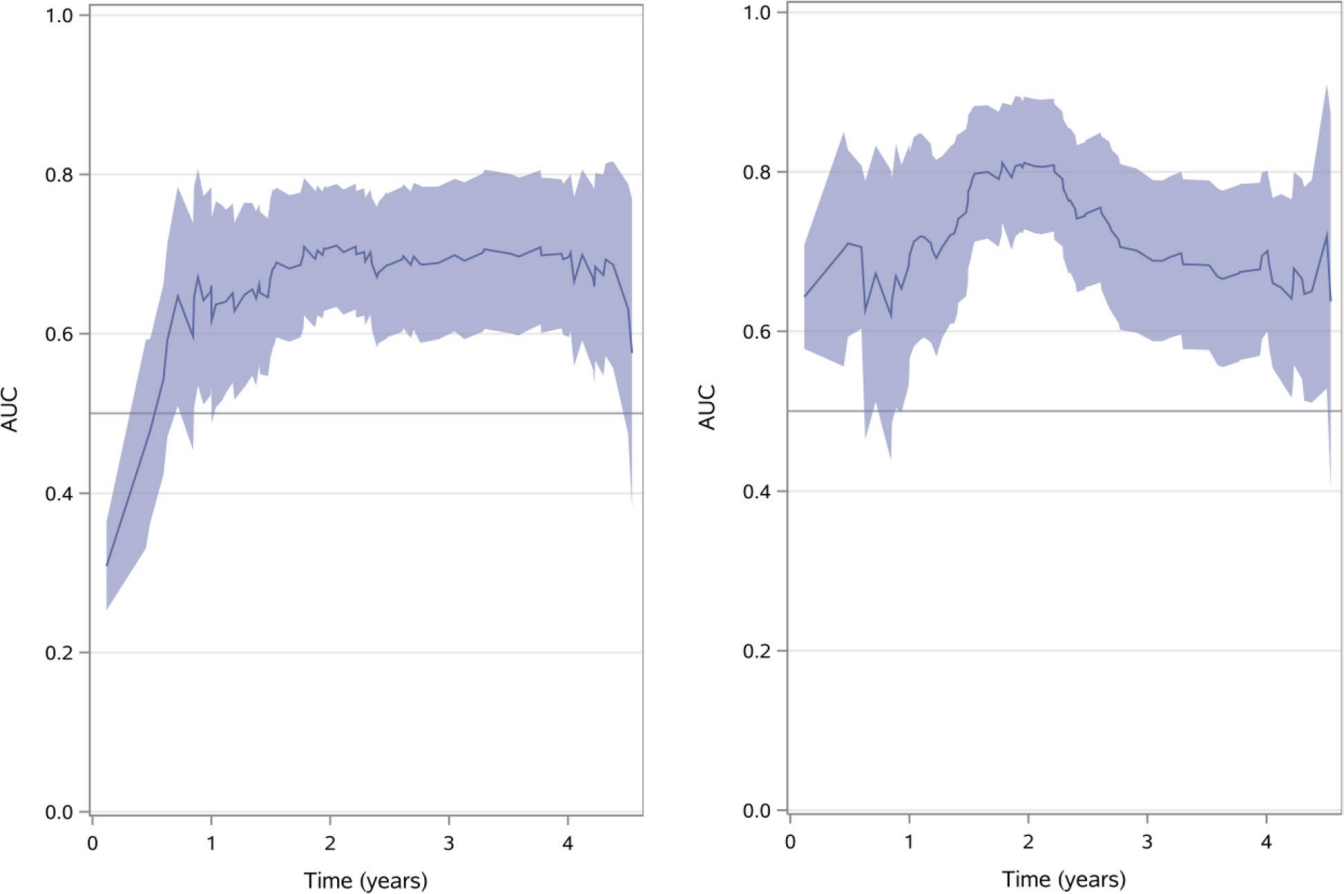
Time-Dependent area under the curve (AUC) with 95% confidence limits for number of months/10 s (left) and number of animals/10 s (right)

Figure 3 shows time-dependent area under the ROC curve:s **(**AUC:s), presented for TUGdt-MB words/time (left) and TUGdt-NA words/time (right). For TUGdt-MB words/time AUC is approximately 0.7 from 2 years and onwards while for TUGdt-NA words/time AUC reaches a maximum of approximately 0.8 around 2 years but is lower before and after that time.

Table 3 shows optimal cutoff values for TUGdt-MB words/time, TUGdt-NA words/time, MMSE and verbal fluency test for prediction times 2 and 4 years. Classification to high risk for progression to dementia is defined as at or below the cutoff value. Also, specificity, sensitivity and Youden index associated with the cutoff values are presented. Optimal cutoff values at 2 and 4 years for TUGdt-NA words/time are 4.0 and 4.5, respectively, while for TUGdt-MB words/time these values are 4.6 and 4.7, respectively. The Youden index for TUGdt-NA words/time and TUGdt-MB words/time are high for prediction up to 2 years (0.53 and 0.33, respectively) and lower for prediction up to 4 years (0.29 and 0.26, respectively).

**Table 3:** Optimal cutoffs with associated specificity, sensitivity and youden index (and 95% Confidence Intervals (CI)) for follow-up times 2 and 4 years

Parameter	Time (years)	Cutoff (95% CI)	Specificity (95% CI)	Sensitivity (95% CI)	Youden index^a^ (95% CI)
TUGdt-NA, animals/10 s	2	4.0 (3.1–5.2)	0.74 (0.52–0.93)	0.79 (0.55–0.98)	0.53 (0.40–0.66)
	4	4.5 (3.7–5.4)	0.65 (0.51–0.83)	0.64 (0.51–0.82)	0.29 (0.13–0.44)
TUGdt-MB, months/10 s	2	4.6 (3.3–5.5)	0.62 (0.50–0.80)	0.71 (0.52–0.88)	0.33 (0.17–0.48)
	4	4.7 (3.6–5.6)	0.61 (0.50–0.79)	0.65 (0.51–0.83)	0.26 (0.09–0.42)
MMSE score	2	27 (26–28)	0.72 (0.53–0.87)	0.74 (0.55–0.92)	0.46 (0.32–0.60)
	4	27 (26–28)	0.77 (0.59–0.94)	0.69 (0.51–0.86)	0.46 (0.31–0.59)
Verbal Fluency score	2	17 (16–19)	0.66 (0.54–0.76)	0.84 (0.71–0.95)	0.50 (0.36–0.63)
	4	18 (16–19)	0.66 (0.51–0.81)	0.67 (0.52–0.84)	0.33 (0.17–0.48)

*TUGdt* Timed Up and-Go dual-task, *NA* Naming animals, *MB* Months backwards, *MMSE* Mini Mental State Examination

^a^Youden index = specificity + sensitivity – 1

## Discussion

Novel findings are provided showing that the TUGdt parameter words/time, even after adjustment for age, gender and education, predicts conversion to dementia over a period of five years in participants with SCI or MCI. Among the TUG-related parameters investigated, word/time showed the best predicative capacity, while time scores of TUG and TUGdt as well as TUGdt cost did not generate any significant predictive results. Our results further showed that the step parameter step length during TUG can work for single task item prediction before adjustment for age, gender and education. After correction for multiple testing TUG-MB words/time remained significant in the adjusted model.

The current results also comprise optimal TUGdt cutoffs for predicting dementia at 2- and 4-year follow-up. The sensitivity of the TUGdt cutoffs was high at 2-year follow-up (TUGdt-NA words/time, 0.79; TUGdt-MB words/time, 0.71, reducing respectively to 0.64 and 0.65 at 4-year follow-up). The cutoffs for the TUGdt tests therefore had comparable sensitivity at 2-year follow-up to the cutoffs for the MMSE score (sensitivity 0.74). The high sensitivity of the MMSE score is to be expected, given the score is an important part of the information on which diagnosis is based throughout the memory assessment process. The comparable sensitivity of the TUGdt score is more remarkable, given the assessment was performed independent of the diagnostic process. An advantage of the TUGdt tests is that they capture dual cognitive and mobility decline, while the MMSE mainly assesses language and memory function [43]. More research is required to compare TUGdt test results and MMSE scores for prediction of dementia incidence under similar study conditions.

It is notable that the step parameter results became non-significant after the adjustments due to these variables high correlation with age. This may be explained by the increasing interdependence between mobility and cognition with age, as a greater degree of cognitive monitoring is required to compensate for age-related declines in the sensorimotor system [44].

In the current study 98 patients from 186 with SCI or MCI at baseline converted to dementia (53%) during follow-up time up to 6.1 years. This corresponds well with annual MCI to Alzheimer’s disease (AD) conversion rates of 10–15% reported for patients examined in clinical settings [45, 46]. In our previous study [24] based on the same baseline population and data collection procedures, 51 of 172 participants (30%) converted to dementia, while TUGdt words/time was associated with dementia incidence and improved dementia prediction compared to demographic characteristics and standard cognitive tests alone over a period of 2.5 years, although only in patients younger than 72 years [24]. However, no significant interactions between the TUG test outcomes and age and sex were observed in the current study, though for TUGdt-NA words/time the p-value for interaction with age was 0.06.

The current study has limitations. The validity of the diagnoses at follow-ups may be questioned, since not all participants went through a re-evaluation at the memory clinic. However, to obtain correct diagnosis, all participants’ medical records were reviewed by an experienced geriatrician. Our participants were recruited in university cities and it is possible the educational level of the sample is higher than in the target population, while analysing education level as a dichotomous variable (university educated or not) may have limited how adequately its confounding effect was captured. Though higher education has been found to be associated with improved baseline performance in both verbal fluency and recall in a population of community dwelling older adults, it had no protective effect for cognitive decline, while some evidence exists for a cognitive reserve for verbal fluency [45]. Moreover, there is a risk that factors besides cognitive functioning and on which no data was collected, such as physical capacity, previous injuries, and pain etc., could have influenced gait performance and thus our results. Still, since our strongest outcome measure is word/time, gait capacity is not of crucial importance.

Our results may also be partly affected by reverse causation. Thus, we carried out sensitivity analyses where patients with a shorter follow-up time than one year were excluded. Results were essentially unaltered, indicating that the effect of reverse causation was minimal. The sample size was somewhat limited and after correction for multiple testing only TUGdtMB words/time remained significant in the adjusted model. There is a risk of an inflated Type I error rate for our uncorrected results, while Type II errors, i.e., false negatives, are a concern for the corrected *p* values.

The strengths of our methodology were examined in a systematic review, [46] which indicated that the UDDGait studies showed good quality according to the Newcastle–Ottawa quality assessment scale for nonrandomised studies in meta analyses [47], and highlighted the TUGdt parameter words/time as an excellent discriminator of different levels of cognitive functioning, with a strong association with cognitive impairment [46]. Moreover, using sHR for expressing the increase of hazard suffers less from selection bias with respect to the endpoints chosen and can indicate risks that happen before the endpoint. Comparable presentation of optimal cutoffs for assessment of dementia prediction is rare. We found only one such study, [48] in which the predictive capacity for conversion to dementia based on sociodemographic and clinical factors were retrospectively examined in a sample of 65 persons with MCI. Results showed that more years in education and lower baseline scores on the Cambridge Cognition Examination (CAMCOG) [49] (which included some MMSE items) were associated with progression to dementia, with comparable sensitivity and AUC as for the TUGdt words/time in our study.

To the best of our knowledge, this is the first prospective longitudinal study to present predictive results including optimal cutoff values from joint clinical simultaneous assessments of cognition and mobility, with results presented in terms of the rarely-used parameter words/time, which combine mobility and cognition outcomes. Our study was performed in a memory clinic, and we welcome further research on prediction of conversion to dementia in other settings and among other populations. Our previous research provided age- and gender-specific normative reference values for TUGdt-NA and TUGdt-MB, and showed fair to good levels of reliability for TUGdt-NA and TUGdt-MB words/time results for cognitively healthy adults (50–91 years of age) [50].

In conclusion, the TUGdt words/time parameter shows potential to serve as an easy to administrate and low-cost tool for assessment of risk of conversion to dementia, useful for research and clinical use in memory clinics. The parameters TUGdt-NA and TUGdt-MB words/time may be able to bridge the gap of insufficient evidence for such clinical outcomes [5]. Based on our growing experience of TUGdt assessment procedures for different levels of cognitive functioning, we presume that TUGdt-MB words/time would also be most suitable for clinical use in other settings other than memory clinics, due to the convenient quantification of the results.

### Supplementary Information

**Additional file 1. **Extraction of step parameters from marker free video recordings of Timed Up and Go tests. Set-up for the TUG experiments. Two cameras were used to record the movement task as well as the verbal task.

## Data Availability

The material analysed during the current study is not publicly available due to its content of sensitive personal data. Datasets generated may be available from the principal investigator Anna Cristina Åberg on reasonable request, after ethical considerations.

## Abbreviations

AD: Alzheimer’s disease
AUC: Area under the Curve
CAMCOG: Cambridge Cognition Examination
CI: Confidence interval
dt: Dual Task test
DLB: Dementia with Lewy bodies
FTD: Frontotemporal dementia
MCI: Mild Cognitive Impairment
MMSE: Mini Mental State Examination
HR: Hazard ratio
PDD: Parkinson's disease dementia
SCI: Subjective Cognitive Impairment
sHR: Standardized Hazard Ratio
TUG: Timed Up and Go test
UDDGait: Uppsala-Dalarna Dementia and Gait project
TUGdt: Timed Up-and Go dual-task test
TUGdt-MB: Timed Up-and Go dual-task test-Months Backwards
TUGdt-NA: Timed Up-and Go dual-task test-Naming Animals
