# Correction: Prediction of conversion to dementia disorders based on timed up and go dual-task test verbal and motor outcomes: a five-year prospective memory-clinic-based study

**DOI:** 10.1186/s12877-023-04427-7

**Published:** 2023-11-02

**Authors:** Anna Cristina Åberg, Johanna R. Petersson, Vilmantas Giedraitis, Kevin J. McKee, Erik Rosendahl, Kjartan Halvorsen, Lars Berglund

**Affiliations:** 1 School of Health and Welfare, Dalarna University, Falun, 791 88 Sweden; 2 Department of Public Health and Caring Sciences, Geriatrics, Uppsala University, Box 564, 52 37, Uppsala, Sweden; 3 Department of Community Medicine and Rehabilitation, Physiotherapy, Umeå University, Umeå, Sweden; 4 Department of Mechatronics, School of Engineering and Sciences, Campus Estado de Mexico, Tecnologico de Monterrey, Atizapan, Mexico, Carretera Lago de Guadalupe Km 3.5, 52926 Atizapan, Estado de Mexico, Mexico

**BMC Geriatrics (2023) 23:535**

10.1186/s12877-023-04262-w

After publication of this article [1], the authors reported that in this article funding information was missing and should have read ‘This work was also funded by Konung Gustaf V:s och Drottning Victorias frimurarestiftelse’.

The original article [1] has been corrected.

## References

[1] Åberg AC, Petersson JR, Giedraitis V (2023). Prediction of conversion to Dementia disorders based on timed up and go dual-task test verbal and motor outcomes: a five-year prospective memory-clinic-based study. BMC Geriatr.

