# Supplementary material for: Prediction of conversion to dementia disorders based on timed up and go dual-task test verbal and motor outcomes: a five-year prospective memory-clinic-based study
Source: BMC Geriatr. 2023 Sep 2;23:535. doi: 10.1186/s12877-023-04262-w (PMC10475186; doi:10.1186/s12877-023-04262-w)
Supplement: Supplementary file 1 — Additional file 1. Extraction of step parameters from marker free video recordings of Timed Up and Go tests. Set-up for the TUG experiments. Two cameras were used to record the movement task as well as the verbal task. [file 12877_2023_4262_MOESM1_ESM.docx]

**Extraction of step parameters from marker free video recordings of Timed Up and Go tests**

Data processing for the gait parameters was based on the documentation of the tests using two high-definition (1080p) video cameras with 81 degrees horizontal field of view and a frame-rate of 25 frames per second (Sony NEX-5T). The cameras were placed on tripods at approximately 1m height. One camera was placed 2m in front of the 3m-line where the participant turned (front view), and the other camera was placed 4m to the side (side view, see figure). The cameras were synchronized by a digital clock visible in both cameras. Calibration of the cameras was done using the known position of at least five points on the floor. These points were marked with black tape. The coordinate system on the floor had one coordinate axis (x) pointing from the chair to the 3m-line, and one coordinate axis (y) pointing perpendicular to the x-axis and to the left as seen when sitting on the chair. For each video camera a mapping from the image-plane to the floor-plane was estimated using the floor markings in the image^1^. From the floor coordinates of the heels at heel-strike, the length and the width of each step was calculated. The procedure is described in more detail elsewhere, ^2,3^, where inter- and intra-rater reliability as well as validity of the method were established.


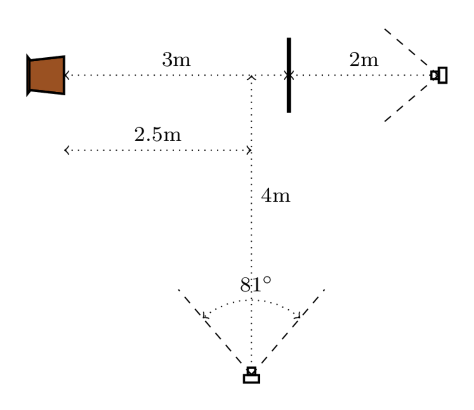


**Set-up for the TUG experiments. Two cameras were used to record the movement task as well as the verbal task**

Each video recording was split into a sequence of images using ffmpeg (https://ffmpeg.org) ^1^ and subsequently processed with OpenPose ^1^ to obtain 25 body keypoints. The trajectories of the keypoints on the feet served to extract only the parts of the video containing gait, and to give an initial estimate of the heel-strike event in the gait sequences. The approximate heel-strike event was corrected by visual inspection of a sequence (strip) of video-frames centered about the approximate heel-strike event. The definition of the heel-strike used was the first frame with visible contact between the foot and ground and where there is a visible plantar-flexion of the foot with respect to the previous frame. The toe-off event was also identified from a sequence of images using the following definition: the time of toe-off is the last frame before the foot loses contact with the floor and where there is still a visible bend or deformation of the toe-part of the shoe. The step parameters step duration (heel-strike to contra-lateral heel-strike), double-stance duration (heel-strike to toe-off) and single-stance duration (toe-off to contra-lateral heel-strike) were calculated from the identified events. Images at the heel-strike instant were cropped and magnified to show the foot only, and the most posterior point (side view) or most lateral point (front view) of the heel in contact with the floor was manually marked. The image location of the marked heel-point was transformed to a point on the floor using the image-to-floor mapping previously determined, from which step length and step width could be calculated.

1. Cao Z, Hidalgo G, Simon T, Wei SE, Sheikh Y. OpenPose: Realtime Multi-Person 2D Pose Estimation

sing Part Affinity Fields. *IEEE Trans Pattern Anal Mach Intell.* 2021;43(1):172-186.

2. Åberg AC, Olsson F, Ahman HB, et al. Corrigendum to "Extraction of gait parameters from marker-free

video recordings of timed up-and-go tests: Validity, inter- and intra-rater reliability" [Gait Posture 90 (2021)

489-495]. *Gait Posture.* 2022;94:195-197.

3. Åberg AC, Olsson F, Åhman HB, et al. Extraction of gait parameters from marker-free video recordings

of Timed Up-and-Go tests: Validity, inter- and intra-rater reliability. *Gait Posture.* 2021;90:489-495.
